# Quality of life in patients treated with radiochemotherapy for primary diagnosis of anal cancer

**DOI:** 10.1038/s41598-022-08525-1

**Published:** 2022-03-15

**Authors:** Christina Sauter, Jan C. Peeken, Kai Borm, Christian Diehl, Stefan Münch, Stephanie E. Combs, Hendrik Dapper

**Affiliations:** 1 Department of Radiation Oncology, Klinikum rechts der Isar, TU München, Ismaninger Str. 22, 81675 Munich, Germany; 2 Deutsches Konsortium für Translationale Krebsforschung (DKTK), Partner Site Munich, Munich, Germany; 3 Institute for Radiation Medicine (IRM), Helmholtz Zentrum München, Ingolstädter Landstr. 1, Neuherberg, Germany

**Keywords:** Gastrointestinal diseases, Cancer, Cancer therapy, Risk factors, Quality of life

## Abstract

Anal cancer and the related treatment are generally known to affect patients’ quality of life. The aim of this study was to assess self-reported quality of life (QoL) of anal cancer patients after combined radiation and chemotherapy, and to identify patient-, disease-, and therapy-related factors associated with QoL. A total of 94 patients treated with definitive chemoradiation for anal cancer at our institution in the period from 2004 to 2018 were identified from our database. QoL was assessed in the remaining 52 patients using the EORTC QLQ-C30 questionnaire (cancer-specific QoL) and the newly developed anal cancer module QLQ-ANL27 (site-specific QoL). Differences in QoL between anal cancer patients and a German age and sex adjusted reference population were examined. The median follow-up was 71 months (range, 7–176). In the cancer-specific QoL module, the anal cancer cohort presented with significantly lower scores in role (− 12.2 points), emotional (− 6.6 points), and social functioning (− 6.8 points), but higher scores in diarrhea (+ 36.3 points) and constipation (+ 13.3 points) than the German reference population. There were no significant differences in disease- or therapy-related factors, but age greater than 70 years and a follow-up time greater than 71 months had a negative impact on global QoL. As for the site-specific QoL, patients with a tumor relapse showed significantly higher symptom scores than patients with a complete clinical remission in all scales except of micturition frequency. Compared to 3D conformal radiotherapy, IMRT treatment seemed to improve non-stoma bowel function (+ 23.3 points), female sexual functioning (+ 24.2 points), and came along with less scores in the symptom scales pain (− 35.9 points), toilet proximity (− 28.6 points), and cleanliness (− 26.2 points). Most of the functional scores of anal cancer patients were lower compared to the general German population, but did not seem to affect the general QoL. Fatigue, physical, and role functioning had the strongest impact on global QoL causing psychological symptoms as important as physical.

## Introduction

Anal cancer is a relatively rare tumor, compromising about 1.5% of all gastrointestinal malignancies^1,2^. In the United States, an incidence of about 8200 was observed in 2017, which constituted for about 0.5% of all malignant cancer diseases^2^. The rate of incidence is constantly rising. From 1992 to 2011, the incidence rate increased about 2.2% per year. This might have been caused by social and cultural changes, which led to a higher exposition to risk factors, including human papillomavirus^3^. Women were more often affected than men. The highest incidence was in the 7th decade of life, with a median age at death of 76 years^3–5^.

Current guidelines recommend radiation and concomitant chemotherapy as the primary therapy for non-metastatic anal cancer^6^. As chemotherapeutic agents, the combination of mitomycin C (MMC) and 5-fluorouracil (5-FU) or capecitabine is widely used and accepted^7,8^. According to stage, radiation should be performed with a dose of at least 45 Gy (Gy) in fractions of 1.8 Gy, whereas a boost of 9–14 Gy should be added with more advanced tumors and affected lymph nodes^8^. Previously, doses were applied by conformal radiotherapy, but lately, intense-modulated radiotherapy (IMRT) became the standard of care^9^. The 5-year overall survival (OS) ranges between 75 and 79%^10–12^, whereas tumors in T1–T2 stage reach 80–90%, and more advanced local disease (T4) has a significantly worse outcome (OS 50%)^13^. The treatment is generally associated with a high rate of acute and late toxicity, mainly including gastrointestinal symptoms like flatulence, painful defecation, diarrhea or constipation^14–16^, as well as a decrease in sexual function^17–19^. Although the use of patient-reported outcomes has by now become the standard of measuring the quality of life of patients^20^, there still are only a few studies on anal cancer patients’ self-reported long term quality of life and their disease- or therapy-related symptoms affecting QoL^14,17,18,21–23^. Therefore, the EORTC (European Organization for Research and Treatment of Cancer) QLQ-ANL27 was recently developed to query typical complications of anal cancer and its therapy^15^.

The aim of this study was to assess self-reported long-term QoL of anal cancer patients after concomitant chemotherapy and radiation and to identify patient-, disease- and therapy-related factors associated with QoL.

## Patients and methods

### Patients

A total of 94 patients with histologically proven invasive carcinoma of the anal canal were treated at our institution in the period from 2004 to 2018. Of these patients, 18 had died, 16 were not reachable, and 8 were not able to fill in the questionnaire because of lacks of language skills or dementia. In the period from October 2018 to March 2019, the remaining 52 patients were called by phone and asked to complete the questionnaires regarding their QoL.

Of these 52 patients, 38 were female and 14 were male. The average age was 64.5 years (48–87). The median follow-up was 71 months (range, 7–176). Pretreatment staging including digital examination, rectoscopy, either magnetic resonance tomography (MRT) or computer tomography (CT) scan of the pelvis, as well as chest and abdominal CT was performed on all patients.

All patients underwent curative radiotherapy (RT) by either IMRT (38) or conformal 3D-technique (14) with or without concomitant chemotherapy.

### Quality of life

All experimental protocols were approved by an Ethikkommission der Technischen Universität München. After obtaining ethic committee’s approval, the outcome was retrospectively analyzed by reviewing medical records and completed with interviews of patients. Long-term QoL was assessed using the EORTC QLQ-C30 and QLQ-ANL27 questionnaires after obtaining permission. The questionnaires were conducted in between October 2018 and March 2019 by telephone interview. Informed consent was obtained from all participants.

All 52 patients were analyzed in respect of the following disease-related factors: tumor size at the time of primary diagnosis (T1/2 vs. T3/4); lymph node status (N0 vs. N+); UICC stage (UICC I/II vs. UICC III/IV); tumor relapse (without tumor relapse vs. tumor relapse); therapy-related factors: radiation technique (IMRT vs. 3D-RT), median applied dose (< 55.8 Gy vs. > 55.8 Gy); patient-related factors: gender (female vs. male), age at QoL assessment (< 70 years vs. > 70 years), length of follow-up period (< 71 months vs. > 71 months).

The 52 patients were asked to participate in the QoL assessment at different times after treatment. QoL was assessed using the European Organization for Research and Treatment of Cancer cancer-specific QLQ-C30 (version 3.0) and the site-specific QLQ-ANL27 questionnaire, which recently had been developed to assess typical complications of anal cancer and its therapy^15,24,25^. The EORTC QLQ-C30 is a highly validated, frequently used questionnaire that includes quality of life for any tumor disease and should be complemented with site-specific questionnaires^25^. It consists of 30 questions that form 5 scales of function (physical, role, emotional, cognitive, social); 3 scales of symptoms (fatigue, nausea or vomiting, pain); 6 single-item scales (dyspnea, insomnia, loss of appetite, constipation, diarrhea, financial difficulties), and a global health-status scale^24^.

Up to now, the QLQ-ANL27 questionnaire is still in phase IV of its development and is currently in a validating and test–retest reliability process^26^. It consists of 27 questions that incorporate 4 multi-item scales to assess bowel function (stoma and non-stoma), pain or discomfort, sexual function (male and female separately), and stoma care. The bowel function scale incorporates questions about the leakage of stools or mucus, and frequent or painful bowel movements. The sexual functioning scale embodies, inter alia, pain during sexual intercourse, affection of sex life through the disease or treatment, and difficulties of erection in men. In addition, 5 single items evaluate frequent urination, keeping clean (need of cleaning oneself more often), proximity to toilet (need to be close to a toilet), lower limb oedema, and planning activities in advance.

As described in the EORTC scoring manual, all scales of the QLQ-C30 and QLQ-ANL27 were linearly transformed, so that all scales range from 0 to 100^27^. A higher scale score represents a higher level of functioning in the 5 (QLQ-C30) and 4 (QLQ-ANL27) scales of function, as well as a higher overall quality of life in the global health-status scale. In the symptom-item and single-item scales a higher level correlates with a higher degree of symptoms/problems for the patients. The calculation of raw-score (RS) and score (S) was implemented according to the EORTC QLQ-C30 and QLQ-ANL-27 scoring manual^27^.

To assess the differences in QoL between healthy people and anal cancer patients, the scores of the EORTC-C30 questionnaire were compared with the data of a German reference population. For that purpose we used a previously published regression model of Schwarz and Hinz^28^. The data of the German reference population was sex- and age-adjusted for this analysis.

### Statistical methods

All statistical analyses were performed with the Statistical Package for Social Sciences software, version 25.0 (SPSS, Chicago, IL). Graphics and tables were created with GraphPad Prism, version 8.1.2 (GraphPad Software, San Diego, CA) and Microsoft Office Word, version 16.15 (Redmond, WA).

Mean values were specified with standard deviation (SD). The QLQ-ANL27 scores were tested with a chi square test. Associations between the QoL scores and study variables were assessed by a Students’ *t* test and Mann–Whitney *U* test according to the nature of the variables. Pearson’s partial correlation coefficients were calculated to assess the association between symptom and function scores. No adjustment was made for multiple comparisons, so p values referred to individual tests rather than a global test for differences. A two-sided p value of < 0.05 was considered statistically significant.

### Ethics approval and consent to participate

The study was performed in accordance with the ethics standards at the Technical University of Munich (TUM) (ethical vote: 385/18s).

Name of committee: Ethikkommission der Technischen Universität München.

## Results

Seven patients (13.5%) did not receive any chemotherapy because of a small tumor size or comorbidities. The majority of patients (71.2%) underwent a chemotherapy consisting of 5-FU (continuous infusion during the first four days and days 29–32 of RT in a dose of 1000 mg/m^2^) and MMC (intravenous bolus on the first and 29th day of RT in a dose of 10 mg/m^2^). Three of these patients suffered from severe side effects and therefore received just one cycle of chemotherapy. Another 8 patients were treated with oral doses of capecitabine (825 mg/m^2^ twice daily during RT). Two of these patients had to stop chemotherapy because of severe leukopenia and hand-foot syndrome.

Seven patients had a stoma, of whom 4 received their stoma because of salvage abdominoperineal resection and 3 others because of toxicity. Two patients (14.3%) of the 3D group and 7 (18.4%) IMRT-treated patients suffered from relapse during the surveillance period.

The median applied total dose at the primary tumor was 55.8 Gy (range, 50.4–60), and the median total dose at the inguinal lymph nodes was 39.6 Gy (range, 36–59.4). In the time from 2008 to 2018, 38 patients received IMRT. Those were treated with a Varian Clinac^®^ DHX linear accelerator (Varian Medical Systems, Palo Alto, CA, USA) or TomoTherapy Hi-ART-System (6 MV) (Accuray, Sunnyvale, USA). Planning and contouring was performed with Treatment Planning System, Eclipse 13.0 (Varian Medical Systems, Palo Alto, CA, USA).

The 14 patients in the 3D-group underwent treatment in the period from 2004 to 2008. All of them were treated with Digital Medical Linear Accelerator from Siemens ONCOR™. Planning was performed with Oncentra MasterPlan software version 3.0 SP1.

For TNM classification, the International Union Against Cancer (UICC) classification was used^29^. Thirty-three patients had T1 or T2 lesions; 19 patients showed T3 or T4 stage; 29 patients did not have any regional lymph node metastases; 21 patients had N+ stage; in 2 patients, lymph node status could not be examined. Twenty-nine patients could be classified into UICC I or II, 21 patients presented in stage III and 2 patients showed distant metastases and were classified into stage IV (Table 1).

**Table 1:** Patient characteristics.

Patient characteristics (n = 52)	n
**Age at time of questionnaire completion (years)**	
Median	64.5
Range	48–87
**Gender**	
Female	38
Male	14
**Months since radiotherapy**	
Median	71
Range	7–176
**T stage**	
T1	10
T2	23
T3	12
T4	7
**N stage**	
N0	29
N+	21
Nx	2
**UICC stage**	
I	9
II	20
III	21
IV	2
**Radiation technique**	
IMRT	38
3D	14
**RT dose**	
Median	55.8 Gy
Range	50.4–60 Gy
**Tumor relapse**	
IMRT	7
3D	2
**Patients with stoma**	7

### EORTC QLQ-C30 questionnaire

**Figure 1:**
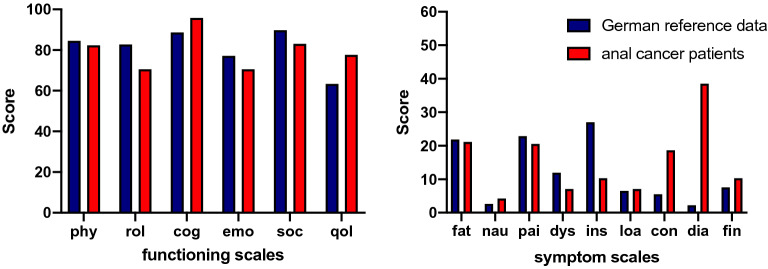
Functioning and symptom scales of the EORTC QLQ-C30 patients vs. German reference population. *phy* physical function; *rol* role function, *cog* cognitive function, *emo* emotional function, *qol* global QoL, *fat* fatigue, *nau* nausea/vomiting, *pai* pain, *dys* dyspnea, *ins* insomnia, *loa* loss of appetite, *con* constipation, *dia* diarrhea, *fin* financial problems.

In comparison to the German reference population, anal cancer patients had a statistically significant reduction in role (70.5, SD ± 22.3 vs. 82.7, SD ± 1.1 points; p = 0.000), emotional (70.5, SD ± 15.0 vs. 77.1, SD ± 0.2 points; p = 0.000), and social functioning (83.0, SD ± 11.4 vs. 89.8, SD ± 1.9 points; p = 0.009), but higher cognitive functioning (95.8, SD ± 12.5 vs. 88.6, SD ± 2.1 points; p = 0.041), as well as overall global health (77.6, SD ± 12.5 vs. 63.4, SD ± 1.3 points; p = 0.000). The physical functioning score did not differ in a statistically significant way from that of the general German reference population (p = 0.365). Most symptom scales and single items did not differ statistically significant either, but the radiation-associated symptoms constipation and diarrhea showed higher scores in anal cancer patients than in the reference group (p = 0.002; p = 0.000) (Fig. 1).

The regarded patient-dependent factors in the anal cancer cohort were gender, age at QoL assessment, and follow-up period. Males showed a better emotional functioning (86.9, SD ± 14 vs. 74.3, SD ± 14 points), patients younger than 70 years seemed to obtain a better physical- (87.2, SD ± 14 vs. 72.2, SD ± 19 points) and role functioning (76.7, SD ± 20 vs. 57.8, SD ± 23 points), as well as a better global QoL (80.2, SD ± 12 vs. 72.1, SD ± 13 points) (p < 0.05). Younger patients and patients with a follow-up period less than 71 months reached lower scores in fatigue (p < 0.05). Except these factors, we could not determine any statistically significant differences in tumor-dependent factors like tumor stage, lymph node status, or UICC stage.

The anal cancer patients were treated with two different radiation techniques: 3D and IMRT. Since the use of 3D conformal radiation was the preferred technique in our center until 2009, there was a huge difference in the patients’ age and their follow-up period. Therefore, we used an age-adjusted matched pair analysis to compare the two subgroups. The 14 3D patients (median age 69 years) were compared with 14 IMRT patients of a similar age (median age 68.5 years). Nevertheless, there was a bias because of the different median follow-up periods which varied between 60 months (range, 20–118) in the IMRT cohort and 152 months (range, 122–176) in the 3D cohort, that could not be balanced. Out of these reasons, the following results should be critically reviewed. Looking at the therapy-dependent factors, IMRT patients showed no significantly different results in the functioning and symptom scales.

Looking at the anal cancer patients, several patient dependent-factors were identified as statistically significant. Patients with an age more than 70 years at the time of QoL assessment showed worse physical (p = 0.002) and role functioning (p = 0.003) as well as global QoL (p = 0.026). The symptom score of fatigue was noticeably higher in patients more than 70 years of age (p = 0.004). All other results are shown in Table 2.

**Table 2:** QLQ-C30 questionnaire: patient-, tumor- and therapy-related factors.

Factors	No.	Physical function	Role function	Emotional function	Social function	Global QoL	Fatigue	Constipation	Diarrhea
**Study population**									
German reference group	52	84.5 (1.2)	82.7 (1.1)	77.1 (0.2)	89.8 (1.9)	63.4 (1.3)	21.8 (2.0)	5.5 (1.3)	2.2 (0.04)
Anal cancer patients	52	82.3 (17)	70.5 (22)*	70.5 (15)*	83.0 (11)*	77.6 (13)*	21.1 (18)	18.6 (30)*	38.5 (19)*
**Tumor stage**									
T1/2	33	86.3 (11)	72.2 (19)	78.5 (15)	80.8 (18)	78.3 (12)	21.2 (17)	17.2 (29)	39.4 (34)
T3/4	19	75.4 (23)	67.5 (27)	76.3 (15)	86.8 (16)	76.3 (13)	21.1 (21)	21.1 (32)	36.8 (33)
**Lymph node status**									
N0	29	84.6 (14)	74.1 (20)	79.9 (16)	81.6 (18)	78.1 (13)	22.6 (17)	13.8 (26)	40.2 (35)
N+	21	80.0 (20)	66.7 (25)	74.6 (13)	84.1 (18)	77.4 (12)	17.5 (20)	23.8 (34)	36.5 (31)
**UICC**									
I/II	29	83.9 (15)	71.8 (20)	7.9 (16)	81.0 (18)	77.6 (13)	24.5 (17)	16.1 (28)	39.0 (36)
III/IV	23	803 (20)	68.8 (25)	75.0 (13)	85.5 (18)	77.5 (12)	16.9 (19)	21.7 (33	37.7 (31)
**Tumor relapse**									
Without relapse	43	82.3 (18)	71.3 (23)	77.5 (15)	83.3 (18)	77.1 (13)	23.5 (19)*	16.3 (30)	38.0 (34)
With relapse	9	82.2 (12)	66.7 (20)	78.7 (15)	81.5 (15)	79.6 (6)	9.9 (13)	29.6 (26)	40.7 (32)
**RT technique**									
IMRT	14	81.1 (14)	70.2 (22)	79.2 (14)	81.0 (20)	77.4 (11)	23.8 (19)	14.3 (22)	47.6 (28)
3D	14	76.2 (20)	64.3 (25)	75.6 (18)	83.3 (19)	69.6 (13)	29.4 (18)	26.2 (40)	45.2 (41)
**Dose**									
< 55.8	27	82.5 (17)	69.1 (22)	78.4 (14)	83.3 (18)	75.6 (14)	21.4 (18)	19.8 (30)	37.0 (35)
> 55.8	21	81.6 (17)	71.4 (23)	75.8 (16)	83.3 (18)	81.0 (10)	21.2 (19)	17.5 (31)	41.3 (33)
**Gender**									
Female	38	82.8 (15)	71.9 (21)	74.3 (14)	83.3 (17)	77.2 (13)	23.1 (19)	17.5 (29)	41.2 (31)
Male	14	81.0 (23)	66.7 (27)	86.9 (14)*	82.1 (20)	78.6 (11)	15.9 (17)	21.4 (34)	31.0 (38)
**Age at QoL assessment**									
< 70 years		87.2 (14)	76.7 (20)	78.1 (15)	81.9 (16)	80.2 (12)	16.2 (17)	20.0 (30)	37.1 (32)
> 70 years		72.2 (19)*	57.8 (23)*	77.0 (16)	85.3 (20)	72.1 (13)*	31.4 (17)*	15.7 (31)	41.2 (36)
**Follow-up period**									
< 71 months	27	84.9 (17)	70.4 (23)	81.2 (15)	82.1 (18)	80.9 (12)	16.0 (19)	12.3 (25)	32.1 (31)
> 71 months	25	79.5 (16)	70.7 (22)	74.0 (15)	84.0 (18)	74.0 (13)*	26.7 (17)*	25.2 (34)	45.3 (35)

*Statistically significant.

Standard deviation inside the parentheses.

Moreover, we examined the questionnaire under the aspect of correlation. Patients reporting high symptom scores generally tended to report lower functioning scores and vice versa. This means, for instance, that patients with a higher level of pain tend to report a lower functioning in the scale of bowel function.

Herein, we found a significantly positive correlation between physical- (r = 0.552) and role functioning (r = 0.559) and global QoL, which suggests, that these factors have the strongest impact on global QoL. Fatigue had the strongest impact on an impaired QLQ-C30 global QoL score (r = − 0.447).

### EORTC QLQ-ANL27 questionnaire

In the functioning scales, patients showed significantly high scores in bowel function without stoma (75.4 points; p = 0.001), and in female sexual functioning (73.8 points; p = 0.0015). Main symptoms were micturition frequency (21.8 points; p = 0.000), leg edema (26.3 points; p = 0.044), toilet proximity (25.6 points; p = 0.000), cleanliness (26.9 points, p = 0.000), and planning activities (21.8 points; p = 0.000).

#### Disease-related factors

Regarding T-stage at the time of primary diagnosis, functioning scores did not show any significant differences. The symptoms pain and leg edema seemed to occur more often in patients with advanced T-stages (T1/2: 19.7 points vs. T3/4: 31.0 points, p = 0.027; and T1/2: 21.2 points vs. T3/4: 36.8 points, p = 0.015).

Neither the UICC stage nor the lymph node status at diagnosis showed any significant differences between UICC stage I/II and III/IV or nodal status (N0 and N+).

Patients with recurrent disease during the follow-up period had a significantly lower bowel function, if they had a stoma because of their treatment (p = 0.049). The other functioning scores did not show any notable differences. All symptom scales except micturition frequency showed lower levels in patients with a complete remission throughout the follow-up period compared to patients with tumor relapse: pain (p = 0.000), stoma care (p = 0.004), leg edema (p = 0.002), toilet proximity (p = 0.000), cleanliness (p = 0.011), and planning activities (p = 0.007).

#### Therapy-related factors

Following our approach in the core questionnaire, we also used an age-adjusted matched pair analysis to compare the two subgroups. The functioning scales of the IMRT group showed a significantly better non-stoma bowel function (83.5 points) than the 3D group (57.3 points) (p = 0.001). Besides, female sexual functioning scores of the IMRT-treated patients were 24 percentage points higher (79.6 vs. 55.6 points; p = 0.000). Regarding the symptom- and item scales, IMRT patients suffered less from pain (16.4 vs. 38.9 points; p = 0.021), toilet proximity (15.6 vs. 47.6 points; p = 0.009), and planning activities (12.6 vs. 42.9 points; p = 0.003).

Looking at the median applied dose (< 55.8 Gy vs. > 55.8 Gy), female patients with a dose higher than 55.8 Gy oddly seemed to have a better-preserved sexual function (p = 0.035), whereas male sexual functioning did not significantly differ.

The only symptom scale that showed significant results was toilet proximity, in which patients with higher radiation doses unexpectedly suffered less (17.5 points vs. 33.3 points; p = 0.045). The other symptom scales did not show any significant difference regarding the applied dose.

#### Patient-related factors

In the QLQ-ANL27 questionnaire, gender could not be identified as a prognostic factor.

According to age, patients were divided into two different groups, older and younger than 70 years of age. None of the functioning scales showed notable differences preferring the younger group, but older patients significantly suffered more often from pain (< 70 years: 19.4 points; > 70 years: 33.0 points; p = 0.009).

Generally, the duration of the follow-up period made a difference in the most functioning and symptom scales. In the functioning scales, non-stoma bowel function and female sexual functioning scores ranged higher in patients with a follow-up period lower than 71 months (non-stoma bowel function: 82.6 points vs. 67.9; p = 0.006; female sexual functioning: 80.4 points vs. 67.8 points; p = 0.006) compared to patients with a follow-up period longer than 71 months.

Accordingly, all symptom scores but micturition frequency and toilet proximity were significantly lower in patients with a shorter follow-up period (p < 0.05). Because of a small sample size (n = 7) the bowel function with stoma was excluded of the table (Table 3).

**Table 3:** QLQ-ANL27 questionnaire: patient-, tumor- and therapy-related factors.

Factors	No.	Bowel function non-stoma	Sexual function male	Sexual function female	Pain	Micturition frequency	Leg oedema	Toilet proximity	Cleanliness	Planning activities
**Tumor stage**										
T1/2	33	78.3 (16)	63.0 (13)	75.2 (15)	19.7 (17)	20.2 (22)	21.2 (17)	22.2 (26)	27.3 (27)	18.2 (25)
T3/4	19	69.0 (19)	66.7 (17)	70.8 (11)	31.0 (17)*	31.6 (32)	36.8 (25)*	31.6 (32)	26.3 (31)	28.1 (20)
**Lymph node status**										
N0	29	75.3 (18)	66.7 (14)	71.0 (17)	23.3 (18)	18.3 (24)	25.8 (24)	24.7 (30)	30.1 (26)	23.7 (26)
N+	21	75.7 (16)	61.9 (14)	78.2 (6)	24.6 (18)	27.0 (33)	27.0 (33)	27.0 (25)	22.2 (30)	19.0 (20)
**UICC**										
I/II	29	75.1 (19)	66.7 (14)	70.8 (17)	22.6 (19)	19.5 (24)	24.1 (23)	23.0 (27)	31.0 (26)	21.8 (26)
III/IV	23	75.9 (16)	61.9 (14)	77.4 (7)	25.4 (17)	24.6 (32)	29.0 (25)	29.9 (25)	21.7 (29)	21.7 (22)
**Tumor relapse**										
Without relapse	43	76.7 (18)	66.7 (13)	75.1 (15)	20.3 (18)	18.6 (26)	21.7 (23)	19.4 (25)	22.5 (27)	17.8 (23)
With relapse	9	62.5 (5)	58.3 (18)	65.6 (7)	40.7 (7)*	37.0 (35)	48.2 (18)*	55.6 (24)*	48.2 (24)*	40.7 (15)*
**RT technique**										
IMRT	14	80.6 (11)	58.3 (8)	79.8 (6)	23.0 (17)	21.4 (31)	28.6 (26)	19.0 (25)	28.6 (32)	16.7 (17)
3D	14	57.3 (20)*	61.7 (20)	55.6 (16)*	38.9 (19)	35.7 (29)	42.9 (25)	47.6 (32)*	40.5 (27)	42.9 (24)*
**Dose**										
< 55.8	27	74.9 (16)	60.4 (14)	68.4 (17)	24.7 (19)	22.2 (32)	27.2 (25)	33.3 (31)	28.4 (22)	23.5 (27)
> 55.8	21	77.8 (15)	70.8 (16)	78.5 (6)*	23.3 (14)	20.6 (22)	28.6 (24)	17.5 (23)*	25.4 (31)	22.2 (19)
**Gender**										
Female	38	75.8 (17)	–	–	23.4 (18)	18.4 (25)	27.2 (24)	27.2 (30)	25.4 (29)	20.2 (21)
Male	14	74.5 (20)			25.0 (19)	31.0 (33)	23.8 (24)	21.4 (25)	31.0 (24)	26.2 (30)
**Age at QoL assessment**										
< 70 years	35	79.7 (12)	67.7 (17)	74.6 (13)	19.4 (17)	19.1 (26)	24.8 (25)	23.8 (29)	22.9 (23)	19.1 (22)
> 70 years	17	67.7 (22)*	60.7 (9)	71.7 (18)	33.0 (18)*	27.5 (32)	29.4 (23)	29.4 (29)	35.3 (34)	27.5 (27)
**Follow-up period**										
< 71 months	27	82.6 (11)	66.7 (14)	80.4 (8)	16.5 (15)	18.5 (27)	17.3 (19)	21.0 (26)	18.5 (23)	13.6 (17)
> 71 months	25	67.9 (20)*	61.1 (16)	67.8 (16)*	31.8 (17)*	25.3 (29)	36.0 (25)*	30.7 (30)	36.0 (30)*	30.7 (27)*

*Statistically significant.

Standard deviation inside the parentheses.

Moreover, the QLQ-ANL27 questionnaire was examined on internal correlation, too. Herein we found a significant negative correlation between the non-stoma- and stoma bowel function with pain (r = − 0.828; and r = − 0.846) and with female sexual functioning (r = − 0.684). The planning activities-score correlated significantly positively with pain (r = 0.626), toilet proximity (r = 0.608), micturition frequency (r = 0.335), cleanliness (r = 0.444) and leg edema (r = 0.541).

## Discussion

This study was performed to assess the quality of life of anal cancer patients after primary irradiation with or without concomitant chemotherapy. With a median age of 64.5 years, our patient cohort did not differ from the epidemiologic data of anal cancer patients in Germany at primary diagnosis. Moreover, in our cohort, sex distribution showed a higher incidence of anal cancer in women (69.2%) than in men, analogous to the epidemiological data^4^. The median follow-up of 71 months (range, 7–176) of our cohort is similar to comparable analyses (Fakhrian et al. 2013: median follow-up 68 months (range, 9–222)^18^; Bentzen et al. 2013: median follow-up 66 months (range, 5–112)^19^).

As primary therapy, our patients were irradiated with a median dose of 55.8 Gy (range, 50.4–60) in combination with a dose of 5-FU/MMC or capecitabine/MMC. The patients of Bentzen et al., as well as those of Welzel et al. received a similar regime with a median dose of 54 Gy (range, 38–66) and 50.4 Gy (range, 43.2–59.4) with a combined chemotherapy^19,30^. In our study 55.8% of the patients presented in UICC stage I or II. In the study of Fakhrian et al., the appropriate group made up a higher proportion (83%)^18^.

### EORTC QLQ-C30 questionnaire

Many authors compared anal cancer patients with a control group. In a comparable Norwegian cross-sectional study, the score of the global QoL of a Danish control cohort was 83, whereas it reached a score of 68 in the group of anal cancer patients^19^. Other global QoL scores varied between 60 and 72 points^14^. The global QoL of our patients presented noticeably higher with a score of 77.6, which might have been due to the large difference in cognitive functioning in our cohort (95.8) compared to that in other studies (76–85)^14^.

Deficits in role, emotional, cognitive, and social functioning, however, could mainly persist more than a decade after the cancer diagnosis^31^. One reason for the deviation of our results from previous comparable studies might be the assessment of the questionnaire. In our study, patients received a lot of attention during the telephone interview, which might not only have led to higher wellbeing, but also might have prompted patients to reveal their functional deficits more accurately.

Other functioning scales of our patients significantly differed from the scores of the German reference population and consequently matched the results of a systemic literature analysis of Sterner et al., who assessed a general impairment in the functioning scales of anal cancer patients compared to healthy patients^14^.

In longitudinal studies of quality of life in anal cancer using the QLQ-C30 questionnaire before, immediately after, and 1 year after therapy, severe impairment in quality of life was observed after treatment. After 1 year, the perceived QoL of anal cancer patients seemed to increase substantially, when scores became almost consistently better than pretreatment^21,23^. These results indicated a huge impairment especially through treatment, that could be justified not only by prognostic factors, but also by a subjective improvement of QoL in comparison to the pretreatment situation. Although the physical functioning did not seem to be much impaired, psychic functioning (role, emotional, social) was strongly affected.

Depression, pain, and fatigue were highly prevalent in cancer survivors, even in the long-term follow-up. Especially fatigue is named as one of the most prevalent and worst symptoms and was observed in 50–100% of cancer survivors^32^. The review of Bloom et al. of long-term QoL of cancer patients confirmed these enormous deficits in these functions. In comparison to a healthy cohort, cancer patients suffered more often from depression and other issues regarding their emotional wellbeing^33^.

Therefore, besides symptom control, psychological aspects play an important role in cancer aftercare. Psychooncological support, depression screening, and social or spiritual aid should be provided during treatment and follow-up to assess secondary psychic diseases early and treat them accordingly.

The symptom scales showed a 70% lower scale score in constipation (p = 0.002), and a 94% lower scale score in diarrhea (p = 0.000). Especially, Welzel et al.’s study in 2011 with 52 German patients showed similar results regarding gastrointestinal symptoms like constipation and diarrhea with scores of 17 and 37 points (our patients: 19 and 39)^30^ (Table 3). Generally, our cohort showed a notable tendency to quality of life impairing gastrointestinal symptoms, which Sterner et al. had described before^14^. Chronic diarrhea was described in 13–50% of anal cancer patients, even in a decade after therapy. This implied a high impact on everyday life with a negative effect on social activities and overall QoL^34^.

We also compared the results of the QLQ-C30 questionnaire regarding the radiation technique. We did not find any significant differences between the matched-pair analysis of the IMRT and 3D study group. However, one has to note that median follow-up of 60 months (range, 20–118) of our IMRT treated patients was substantially shorter than the follow-up of the 3D patients with 152 months (range, 122–176).

The shorter period between end of therapy and questionnaire assessment might have caused a too high estimation in the functioning scales and a too low in the symptom scores, since possible symptoms might not yet have been evident. As already described, using IMRT technique reduced small bowel acute toxicity by application of lower doses in other IMRT-3D comparation studies^35^. This suggested, that using IMRT could lead to a better long-term toxicity. Due to a small number of conventionally treated patients, a significant statement was not possible, though.

The global QoL was significantly higher in the group of patients younger than 70 years than in the group of patients older than 70 years (p = 0.026). This higher valued QoL was reflected in a significantly higher physical and role functioning (p = 0.002 and p = 0.003), too.

In addition to empirical findings, Schwarz and Hinz's analysis also described a general tendency towards lower functioning in everyday life and higher impairment among older people^28^.

Interestingly, there were no relevant differences in the cognitive, emotional and social functioning. Even the gastrointestinal symptoms seemed to be nearly as severe. Allal et al., who parted their patients into groups with a cut at 71 years of age, could not find any significant differences regarding age, except in a reduced physical functioning in the older group (85 vs. 73 points, p = 0.08)^36^. While older patients suffered from more comorbidities and were subject to physiological aging processes, the symptom load seemed to be more severe and stigmatizing for younger patients^37,38^. So younger patients subjectively perceived more impairment despite objectively better functioning. A greater difference in the scores was observed for fatigue (< 70 years: 16.2, > 70 years: 31.4; p = 0.004). Age-related physical reduction and comorbidities might be discussed as a reason for the ongoing fatigue in patients over 70 years, even years after cancer therapy, what leads to a vicious circle of inactivity causing intensification of fatigue, especially in the elderly^39,40^.

### EORTC QLQ-ANL27 questionnaire

As site-specific questionnaire, the QLQ-ANL27 was lately developed in collaboration of professionals and patients of different countries. The use of “patient-reported-outcomes” (PROs) as a measuring instrument for the QoL of patients now is the gold standard^20^. So far, the use of PROs in anal cancer patients was inconsistent and questionnaires of other tumor-sites like the EORTC QLQ-CR29 colorectal-specific questionnaire^41^ were used. The QLQ-ANL27 now is in Phase IV of its development and is currently validated in international studies^42^.

In the functioning scales our patients showed a noticeably better non-stoma bowel function (75.4 vs. 68.3 points), but the bowel function generally seemed to be impaired. Associated symptoms like flatulence, frequent and painful defecation, urgent and unintended stool were named as common symptoms in anal cancer patients in the secondary literature^14,15^. A review of Pan et al. demonstrated chronic gastrointestinal symptoms after irradiation of anal cancer in 7–64.5% of all patients^16^. The pathophysiologic processes are not yet fully understood. It is assumed that radiation inducts cytokine cascades, which possibly persist for decades and lead to edema, inflammatory processes up to ulceration and fibrosis^43^. These pathophysiologic changes may in turn promote increased gastrointestinal transit^44,45^. Chronic motility disorders are mentioned as the most important cause for excessive bacterial bowel colonization. Other reasons for the impairment of gastrointestinal functioning are malabsorption, chronic inflammatory bowel disease, as well as structural changes^43^.

While our female patients presented more frequently with problems like dyspareunia, vaginal dryness and stenosis, erectile dysfunction (ED) and impotence were a frequent matter for our male patients. In other reviews, these complaints were also observed as early and late complications of anal cancer and its therapy. Up to 60% of women complained about dyspareunia^17,18^ and about 60–71% of men about erectile dysfunction, while just 20% of healthy men suffered from ED at the same age^17–19^. Because of the anatomical proximity to the primary tumor and the consequently high radiation dose and use of chemotherapy, these results could be compared with the sexual function after therapy of gynecologic cancers^46^. As a side effect of the therapy, women may suffer from a decrease in estrogen production or nerve damaging, what may result in vaginal dryness, bleeding and itching, up to vaginal stenosis, leading to discomfort and pain during sexual intercourse^18,47^. In the secondary literature, the prevalence of ED was 20–40% in patients at the age of 60–69 years, in patients over 70 years even 50–100%. This implicated that our patients with a median age of 68 years (range, 48–85) probably had erection problems before therapy, yet^48^. Nevertheless, radiogenic factors may play a role in the development of ED, too. The female sexual functioning (73.8 points) showed better maintenance in comparison to the male sexual functioning (64.6 points). This observation was similar to the statement in a study of Allal et al., where males reported a higher impairment of their sexual functioning^36^.

Comparing the radiation techniques IMRT and 3D, the IMRT cohort presented with a higher preserved bowel function in the non-stoma patients (p = 0.001), and a noticeably better-preserved sexual functioning in females (p = 0.000). As already described in the literature, patients seemed to benefit extremely from the application of a tissue- and organ-at-risk sparing technique such as IMRT, concerning late toxicities^49,50^. The reason for the higher late toxicity of 3D conformal radiotherapy could be the higher dose volume of the surrounding tissue and organs at risk, which was significantly reduced by the IMRT technique^51^.

The specific problems of anal cancer patients like pain, toilet proximity, and planning activities have been hardly recorded in former questionnaires but were less severe in our IMRT-treated cohort. These symptoms determined a severe impairment in the patient-related quality of life^15^. Despite these promising results in favor of the IMRT, results should be evaluated critically. The follow-up of the two cohorts differ significantly with 60 months (range, 20–118) in the IMRT group and 152 months (range, 122–176) in the 3D group. The longer follow-up period in this group allowed a longer period of symptom development. In the 3D cohort, the bowel function in the patients with stoma was better than in the patients without stoma. Wearing a stoma was generally perceived as stigmatization, but also could be relieving in case of severe symptoms. Although colostomy-free survival has been used as a clinical endpoint and thus as a measurement tool for the success of therapy, surgery should be considered as a possible treatment in patients with severe gastrointestinal symptoms^14,52^.

Regarding the age-related quality of life, younger patients tended to better results. Gastrointestinal symptoms like constipation are a general problem in the elderly. In several longitudinal studies, a deterioration of the symptoms diarrhea^21^ and constipation^21,23,53^ was observed in all patients and regardless of age in anal cancer treatment. This suggested age as an additional independent risk factor, but not as cause of diarrhea and constipation.

Depending on no clinical remission after therapy or recurrence during the follow-up period, our cohort showed significantly higher scores in almost all symptom scales. Failure to achieve complete remission or recurrence after primary therapy is followed by burdensome therapies, such as abdominoperineal rectal extirpation (APR) with permanent colostomy, re-irradiation, or chemotherapy with tremendous toxicities, thus placing a new burden on patients. In the current literature, a morbidity of 35% after APR is described^54^, which is defined by gastrointestinal symptoms, wound healing disorder, and pain. In our study, patients with recurrence reached a score twice as high as patients with a complete remission in pain (p = 0.000). Re-irradiation of sensitive regions might have led to gastrointestinal symptoms, micturition problems, and sexual disorders, too^26^. Generally, radiation doses higher than 60 Gy could lead to late toxicities in about 37% of the patients, lower doses in about 14%. Gastrointestinal symptoms also were significantly more frequent (p = 0.001)^16,55,56^. For this reason, there is an essential need for newer treatment options, particularly in advanced stages of anal cancer, to allow patients to have an adequate quality of life.

## Limitations of our study

There are some limitations of our study. The first limitation is the retrospective character of the study and its small sample size. The questionnaire was assessed at very different time points after therapy without determined dates. Also, the interview was conducted by phone and not anonymously sent out by mail.

## Conclusion

Most of the functional scores of anal cancer patients were lower compared to those of the general German population, but did not seem to affect the general QoL. Fatigue, physical complaints, and role behaviors had the strongest influence on global quality of life, so psychological symptoms were as important as physical symptoms. Anal cancer patients suffer most of all from gastrointestinal symptoms, as diarrhea and constipation, that are associated with pain, toilet proximity or the need of planning activities. Patient reported outcomes should be used in long-term studies to further address the symptoms of anal cancer patients and their special needs.

## Data Availability

The present data are summarized in this paper (METHODS). The complete dataset can be obtained from the authors by interested readers upon formal request.

## Abbreviations

APR: Abdominoperineal rectum extirpation
CBCT: Cone-beam-CT scan
CT: Computed tomography
ED: Erectile dysfunction
EORTC: European Organization for Research and Treatment of Cancer
5-FU: 5-Fluorouracil
Gy: Gray
IGRT: Image-guided radiotherapy
IMRT: Intensity modulated radiotherapy
MMC: Mitomycin
MRT: Magnetic resonance tomography
OS: Overall survival
PRO: Patient reported outcome
QLQ: Quality of life questionnaire
QoL: Quality of life
RS: Raw score
RT: Radiotherapy
S: Score
UICC: Union Internationale Contre le Cancer
